# New Spectral Index for Detecting Wheat Yellow Rust Using Sentinel-2 Multispectral Imagery

**DOI:** 10.3390/s18030868

**Published:** 2018-03-15

**Authors:** Qiong Zheng, Wenjiang Huang, Ximin Cui, Yue Shi, Linyi Liu

**Affiliations:** 1 College of Geoscience and Surveying Engineering, China University of Mining and Technology (Beijing), Beijing 100083, China; zhengqiong@student.cumtb.edu.cn (Q.Z.); cxm@cumtb.edu.cn (X.C.); 2 Key Laboratory of Digital Earth Science, Institute of Remote Sensing and Digital Earth, Chinese Academy of Sciences, Beijing 100094, China; shiyue@radi.ac.cn (Y.S.); liuly35@radi.ac.cn (L.L.); 3 Key Laboratory of Earth Observation, Hainan Province, Sanya 572029, China; 4 University of Chinese Academy of Sciences, Beijing 100049, China

**Keywords:** yellow rust, Sentinel-2 MSI, red edge disease stress index (REDSI), winter wheat, detection

## Abstract

Yellow rust is one of the most destructive diseases for winter wheat and has led to a significant decrease in winter wheat quality and yield. Identifying and monitoring yellow rust is of great importance for guiding agricultural production over large areas. Compared with traditional crop disease discrimination methods, remote sensing technology has proven to be a useful tool for accomplishing such a task at large scale. This study explores the potential of the Sentinel-2 Multispectral Instrument (MSI), a newly launched satellite with refined spatial resolution and three red-edge bands, for discriminating between yellow rust infection severities (i.e., healthy, slight, and severe) in winter wheat. The corresponding simulative multispectral bands for the Sentinel-2 sensor were calculated by the sensor’s relative spectral response (RSR) function based on the in situ hyperspectral data acquired at the canopy level. Three Sentinel-2 spectral bands, including B4 (Red), B5 (Re1), and B7 (Re3), were found to be sensitive bands using the random forest (RF) method. A new multispectral index, the Red Edge Disease Stress Index (REDSI), which consists of these sensitive bands, was proposed to detect yellow rust infection at different severity levels. The overall identification accuracy for REDSI was 84.1% and the kappa coefficient was 0.76. Moreover, REDSI performed better than other commonly used disease spectral indexes for yellow rust discrimination at the canopy scale. The optimal threshold method was adopted for mapping yellow rust infection at regional scales based on realistic Sentinel-2 multispectral image data to further assess REDSI’s ability for yellow rust detection. The overall accuracy was 85.2% and kappa coefficient was 0.67, which was found through validation against a set of field survey data. This study suggests that the Sentinel-2 MSI has the potential for yellow rust discrimination, and the newly proposed REDSI has great robustness and generalized ability for yellow rust detection at canopy and regional scales. Furthermore, our results suggest that the above remote sensing technology can be used to provide scientific guidance for monitoring and precise management of crop diseases and pests.

## 1. Introduction

Wheat is an important grain crop in China and has extraordinary significance for ensuring national food security. Various wheat diseases have become major factors for decreased wheat yields in China [1]. Yellow rust is a type of fungal disease induced by *Puccinia striiformis* f. sp. *tritici* (*Pst*), and is one of the most destructive diseases for wheat [1,2]. It is characterized by a high rate of incidence and harbors epidemic characteristics [3,4]. Yellow rust can cause major yield loss (up to 29.3% of the national total production in China) and impact the resultant quality of wheat [1]. Traditionally, monitoring wheat yellow rust has mainly depended on visual inspection in the field, which has been not only inefficient and time-consuming, but also destructive to the ecological environment when combined with excessive use of pesticides [2,5]. In practice, yellow rust tends to occur sporadically across an area. Therefore, it is necessary to find a more accurate, convenient way to monitor the distribution of the disease in the field.

Remote sensing technology has proved to be an efficient tool for monitoring crop growth and identifying crop diseases over the last several decades. Such diseases include: rust infection [5,6,7,8,9], powdery mildew [10,11], fusarium head blight [12], Russian aphid [13] in wheat, bacterial leaf blight, leaf folder and panicle blast in rice [14,15,16], cyst nematode and iron chlorosis in soybeans [17,18], late blight disease, and xanthomonas perforans in tomatoes [19,20], cercospora leaf spot, pathogens and necrosis in sugar beet [21,22,23], leafroll disease in grapes [24], and leafhopper stress in cotton [25]. It is well known that leaf water, pigment contents, and the internal structure of plants may be changed when infected with disease, and these physiological and biochemical changes are reflected in its spectral signature (i.e., the variation in reflectance magnitude and change in spectral shape). A number of studies have been proposed for the use of sensitive original bands or vegetation indexes (VIs) that identify and monitor crop diseases at leaf and canopy scales. For instance, Moshou et al. [4] found that the most sensitive wavelengths for yellow rust detection were at: 680 nm, 725 nm, and 750 nm. Huang et al. [5,26] used a photochemical reflectance index (PRI) for detecting yellow rust disease at the canopy and field scales (R^2^ = 0.91), and proposed a vegetation index, YRI (yellow rust index), to distinguish yellow rust from healthy wheat as well as from powdery mildew and aphids, respectively. Zhang et al. [27] found that only the physiological reflectance index (PhRI) was sensitive to yellow rust disease at all growth stages. Devadas et al. [7] declared that no spectral index could totally discriminate wheat rust (yellow rust, leaf rust, and stem rust) at the leaf scale, but the anthocyanin reflectance index (ARI) could distinguish yellow rust from healthy wheat. Hyperspectral data is widely used in crop disease detection to capture crop biophysical variations caused by infestations on account of its abundant narrow bands and high spectral resolution.

In recent years, many methods or models have been used to select the sensitive features for crop disease detecting and discriminating between different crop diseases [23,28]. Random forest (RF) algorithm, an ensemble technique, has the ability to produce a variable importance ranking [29]. This information is valuable for e users to select variables to build simpler, more readily interpretable models [30]. Chemura et al. [31] used the RF out-of-bag score to select 4 important bands in coffee leaf rust (CLR) discrimination. Fletcher et al. [32] demonstrated that shortwave-infrared bands were the most important variables in discriminating the pigweeds from soybean using RF. Fisher linear discriminant analysis is a method used to find a linear combination of continuous independent variables which characterize or separate two or more classes of objects and events [33]. It performs better when sample sizes are small. Bajwa et al. [17] indicated that linear discriminant models on spectral reflectance data and VI were able to classify healthy soybean from the diseased with an accuracy of 81–93%. Zhang et al. [34] adopted fisher linear discriminant analysis to discriminate between the three healthy levels (normal, slightly-damaged, and heavily-damaged) of powdery mildew with the extracted SFs. Random forest algorithm and fisher linear discriminant analysis were widely used in the feature selection and disease discrimination, respectively.

Most vegetation indexes/models are established to detect yellow rust diseases based on hyperspectral data collected by hyperspectral sensors (i.e., Hyperion, AVIRIS), hence, these VIs and models can hardly be applied directly for monitoring crop diseases at large scales due to the narrow coverage, high cost, and low availability of hyperspectral data [2,35]. Multispectral satellite images (i.e., high and medium resolutions) have been widely used to monitor crop diseases, and are characterized by wide swath coverage, relatively low cost, and are free of charge. For example, Chen et al. used Landsat multispectral imagery to detect the presence of take-all disease in wheat [36]. Oumar and Mutanga demonstrated that Worldview-2 data had the ability to predict *T. peregrinus* damage in plantation forests [37]. Yuan et al. used Worldview-2 and Landsat 8 data to monitor the spatial distribution of crop diseases and pests at regional scales [38]. However, the problem is the incompatibility between existing hyperspectral features (original bands and common VIs) and current multispectral satellite data.

The Sentinel-2 MSI sensor was launched in 2015 by the European Space Agency (ESA) and was designed to meet the needs of “Global Monitoring for Environment and Security” (GMES) [39]. The newly developed multispectral sensor is a composite of multispectral and hyperspectral sensors and includes refined spatial, spectral, and temporal resolutions, providing important information for precision agriculture [40]. More details for Sentinel-2 MSI are shown in Table 1 [31,41]. Sentinel-2 is free to use and the revisit cycle is 10 days (or 5 days when two satellites are operating simultaneously), which makes it attractive for temporal feature analysis [41]. The sensor achieved refined spatial resolution (10 m and 20 m) and wide swath coverage (290 km). More importantly, compared with generally high-medium resolution satellite data, Sentinel-2 has three red-edge bands at the following central wavelengths: 705 nm (B5), 740 nm (B6), and 783 nm (B7), and the three bands at spatial resolution of 20 m, which provide abundant information for estimating and monitoring the biophysical state of plants. For example, the study by Chemura et al. [31] demonstrated that resampled Sentinel-2 sensor data is able to estimate the infection levels (healthy, moderate, and severe) of coffee leaf rust (CLR) at the leaf level. Fernández-Manso et al. [42] pointed out the red-edge wavelengths for Sentinel-2A were suitable for fire burn severity discrimination, and the red-edge spectral indexes demonstrated an improved performance compared to conventional vegetation indexes for identifying fire burn severity levels. Shoko et al. [43,44] used Sentinel-2 MSI data to discriminate and map C3 and C4 grass species, and the results from Sentinel-2 outperformed Worldview 2 and Landsat 8 images. Nevertheless, according to our literature review, we find that multispectral sensors have seldom been used to monitor yellow rust infection [35], and there are few researches describing the potential of Sentinel-2 MSI for detecting wheat yellow rust disease.

This study used the relative spectral response (RSR) function of the Sentinel-2 MSI sensor and canopy hyperspectral data to simulate the reflectance of Sentinel-2 sensor channels, and explored the potential of the Sentinel-2 MSI sensor for identifying yellow rust infection in winter wheat. The objectives were to: (1) select the most sensitive bands of multispectral data (Sentinel-2) for identifying healthy wheat and both slight and severe yellow rust infection in winter wheat; (2) propose a new red-edge multispectral vegetation index for discriminating yellow-rust-infected winter wheat from healthy wheat; and (3) map yellow rust infection using realistic Sentinel-2 satellite imagery at regional scales.

## 2. Materials and Methods

**Figure 1:**
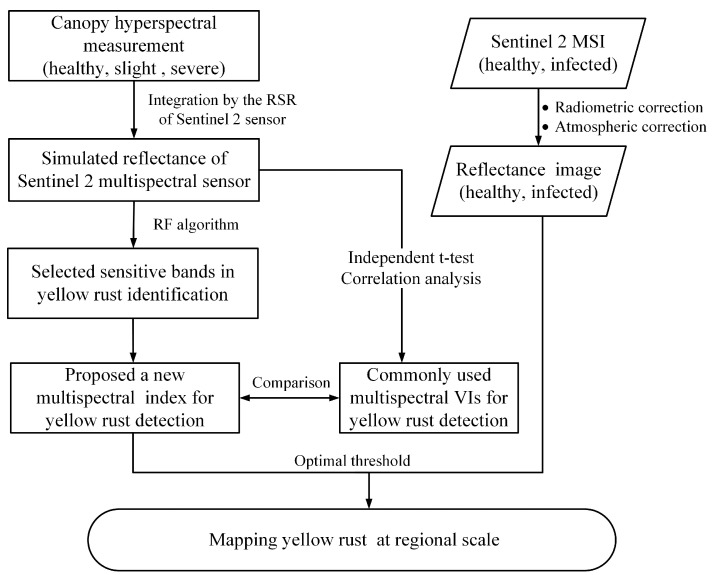
Flowchart of data analysis and processing.

Figure 1 presents a flowchart of yellow rust infection monitoring and identification for winter wheat based on Sentinel-2 satellite data. The hyperspectral data was collected in a canopy experiment and used to simulate the broadband data based on the RSR function of the Sentinel-2 sensor. Simulated broadband data was used to select the most important bands and a new multispectral index was proposed for yellow rust identification. By comparing the availability and superiority of the new index with the commonly used spectral vegetation indexes, the index was applied to actual Sentinel-2 data to complete the detection of yellow rust infection at regional scales using the optimal threshold method.

### 2.1. Study Sites

The canopy scale experiment was carried out at the experimental field of the Chinese Academy of Agricultural Sciences at the winter wheat grain filling stage in 2017. The field is in Langfang (39°30.48′ N, 116°36.14′ E), Hebei Province, China, where the average annual precipitation is 554.9 mm and average annual temperature is 11.9 °C. The soil had a nutrient content in the topsoil layer (0–30 cm depth) of about 1.41–1.47% of organic matter, 20.5–55.8 mg·kg^−1^ of available phosphorus, and 116.6–128.1 mg·kg^−1^ of rapidly available potassium. The winter wheat cultivar known as ‘Mingxian 169’ was selected because of its moderate susceptibility to yellow rust. The yellow rust pathogens infected the winter wheat through an inoculation process (spore solution concentration of 9 mg 100^−1^ mL^−1^) according to the National Plant Protection Standard (NPPS) on 13 April 2017. For the yellow rust experiment group, 39 replicates were planted in a plot with a size of 3 m × 13 m. For the control group, 12 replicates were planted in a plot with a size of 3 m × 6 m. A gradient of disease severity was gradually generated in the field after an inoculation period of 5–7 days. To avoid cross-infection of disease, the distance between each group was no less than 5 m, the control plots had use of pesticides to prevent infections. In this study, each plot was placed under the same cultivation and management conditions (200 kg·ha^−1^ nitrogen and 450 m^3^·ha^−1^ water) to avoid the influence of extraneous factors such as: cultivar, water, fertilizer, and field management. 

**Figure 2:**
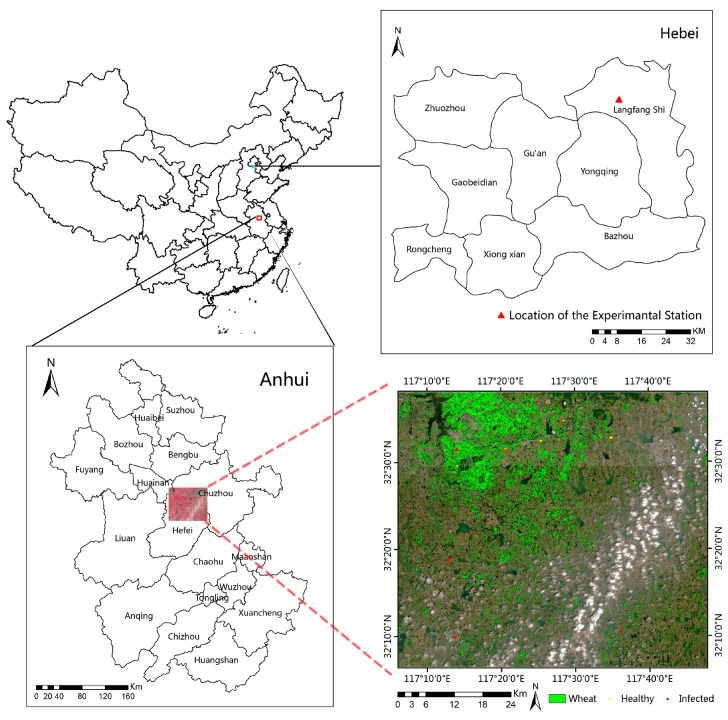
Location of the two study sites.

Field surveys of wheat yellow rust infection were conducted in Chuzhou and Hefei, Anhui Province, China (32°6.36′–32°38.02′ N, 117°6.09′–117°49.10′ E) (Figure 2) on 9 May 2017 at grain filling stage, where winter wheat is considered to be one of the area’s major crops. The average annual temperature of the area is 15 °C and the average annual precipitation is 1000 mm, and 40% to 80% of the precipitation occurs during summer. Low temperature and high humidity provide favorable conditions for yellow rust, so that the disease occurs almost every year in this region during the winter wheat growing season. Therefore, we chose this region as the study area for the regional detection experiment (Figure 2). 

### 2.2. Data Collection

#### 2.2.1. Canopy Spectral Data

The hyperspectral data at the canopy scale were measured using an ASD FieldSpec Pro spectrometer (Analytical Spectral Devices, Inc., Boulder, CO, USA) at grain filling stage on 15, 18, and 25 May 2017. The spectral range of the spectrometer was between 350 nm and 2500 nm, with 3 nm and 10 nm spectral resolution in the 350–1000 nm and 1000–2500 nm regions, respectively. All canopy spectral measurements were taken with a 25° field of view at a height of 1.3 m above the ground. A 40 cm × 40 cm BaSO_4_ calibration panel was performed every 10 measurements to correct the changes in the illumination condition. All experiments were measured under cloudless conditions between 10:00 a.m. and 14:00 p.m. (Beijing local time) when minimum variations in solar view angle occurred. The reflectance spectrum of each sample was measured ten times, and their average was considered as the reflectance spectrum of the samples. The spectral reflectance of 113 winter wheat samples was selected at the canopy scale by eliminating the hyperspectral data affected by the noise, including 29 healthy samples and 84 diseased samples with different severity levels.

#### 2.2.2. Image Acquisition and Field Survey

Two simultaneous Sentinel-2 multispectral images were acquired on 12 May 2017, from https://scihub.copernicus.eu/, and the full coverage image was mosaicked by two images acquired simultaneously. More information about the image is shown in Table 1. Atmospheric correction for the Sentinel-2 image was conducted based on the Sen2cor atmospheric correction toolbox in the Sentinel Application Platform (SNAP) software, and we also resampled the spatial resolution of all bands up to 10 m for subsequent analysis [40].The coastal aerosol (B1), water vapor (B9), and cirrus (B10) were removed from the study due to their irrelevance. We conducted a ground survey on 9 May 2017, and investigated 27 sample plots in the region. The size of each sample point was 10 m × 10 m, and five sampling subplots with a 1 m × 1 m in each plot (five-point sampling) were used to record the average severity [45]. Each plot’s central coordinates were collected using a Trimble GeoXT DGPS with submeter accuracy. 

#### 2.2.3. Assessment of the Disease Index

**Figure 3:**
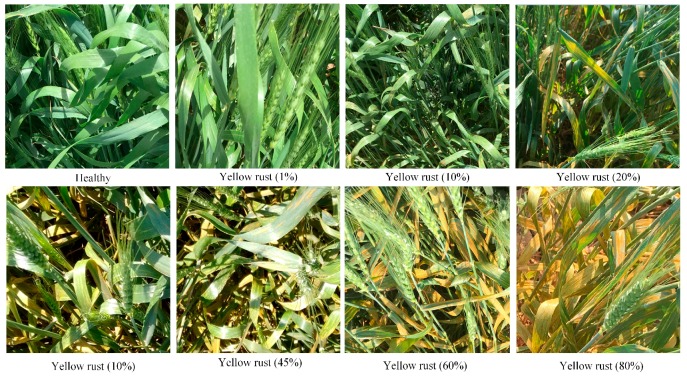
Photos of healthy and different incidences of yellow-rust-infected winter wheat canopies.

The disease index (DI) was used to describe the severity of crop diseases. An assessment of the DI was performed simultaneously with a spectrum acquisition process for wheat yellow rust [5]. A total of 10 individual samples were randomly selected from symmetrical five points of each plot to evaluate the yellow rust incidence level. All tests were conducted by the same individual to eliminate human error. According to the National Rules for the Investigation and Forecasting of Crop Diseases (GB/T 15795-1995), the level of disease incidence (x) is divided into 9 categories (Figure 3): 0%, 1%, 10%, 20%, 30%, 45%, 60%, 80%, and 100%, in which 0% represents no infection and 100% represents the greatest amount of infection [5]. The DI formula is calculated as follows:

(1) $$DI=\frac{\sum xf}{n\sum f}\times 100$$

where x is the value of incidence level, *n* is the value of highest disease severity gradient (*n* = 8), and *f* is the number of leaves for each degree of disease severity.

In addition to the continuous variable DI for evaluating the diseases status, the disease severity of the canopy was also quantitatively classified into three classes for subsequent research and analysis. These include: 29 healthy wheat samples (DI = 0), 39 slight yellow rust infection samples (0 < DI ≤ 20%) and 45 severe yellow rust infection samples (DI > 20%). The criterion for these classifications was suggested by the national plant protection department.

### 2.3. Methods

#### 2.3.1. Simulation of Multispectral Signals Based on Sentinel-2’s RSR

We integrated the field canopy hyperspectral data based on the sensor’s RSR function to simulate the multispectral reflectance of Sentinel-2 (Formula 2) to assess its potential for winter wheat yellow rust monitoring and detection. The formula is given as:

(2) $$R_{sentinel2}=\int_{\lambda_{start}}^{\lambda_{end}}f(x)dx$$

where $R_{sentinel2}$ is the simulated reflectance of the multispectral channel of Sentinel-2 sensor, $\lambda_{start}$ and $\lambda_{end}$ represent the beginning and ending reflectance wavelength of Sentinel-2’s corresponding channel, respectively, and f(x) is the RSR function of the Sentinel-2 sensor.

#### 2.3.2. Using Multispectral Vegetation Indexes for Yellow Rust Detection

Following the integration processing of hyperspectral data, an independent t-test was applied to examine the statistical significance of all bands based on healthy, slightly, and severely infected samples at the highest significance level (*p*-value < 0.001). We found that seven Sentinel-2 spectral bands (i.e., B3, B4, B5, B6, B7, B8, and B8a) manifested excellent potential for discriminating healthy and infected (slight and severe) samples (*p*-value < 0.001) as shown in Table 1. According to the literature review, numerous spectral vegetation indexes (SVIs) have been used to identify crop diseases. An independent t-test was used in this study to explore the significance of the SVIs related to the above seven bands, and the most significant (*p*-value < 0.001) SVIs were selected to identify yellow rust. These SVIs included conventional and red-edge vegetation indexes (as shown in Table 2). The conventional VIs, such as the NDVI, are often used to evaluate crop’s growth state, and RGR has been used to estimate leaf pigments. GNDVI and VARI_green_ have been used to monitor crop disease [46,47,48,49]. The red-edge VIs include the NDVI_re_ which is similar to the NDVI but uses one of the red-edge bands (B5, B6, B7) instead of the red band (B4). NREDI is the index of normalized red-edge bands [42,46,50], and PSRI and ARI indicate crop physiological conditions and leaf pigments, respectively [42,51].

A correlation analysis was performed between SVIs and DI to obtain better identification accuracy for yellow rust, and SVIs whose correlation coefficient was R^2^ < 0.5 were excluded from this study. Finally, only nine SVIs were used for yellow rust detection, including: NDVI, VARI_green_, EVI, RGR, NDVIre1, NREDI1, NREDI2, NREDI3, and PSRI.

#### 2.3.3. Random Forest Algorithm

Random forest (RF) is an ensemble of learning algorithms proposed by Breiman [32]. It consists of a set of independent, unpruned decision trees. RF assumes an original training set with *N* instances, and each instance with *M* attributes. During the forest construction process, “Random” performs in two aspects: (1) It samples a new training set with replacement at each iteration, and the new training set is the same size as the original set; (2) Rather than choosing the best split among all attributes, *m* attributes are randomly chosen from *M* at each node and then these *m* attributes are used to split the node according to the principle of the decision tree algorithm, where *m* << *M*, and it is held constant during the forest construction process. Because the algorithm does not use all samples for model training at one time, this makes it possible to use the remaining samples (out of bag data) to evaluate the out of bag error (OOB error). Moreover, the principle of the feature importance ranking is to compare the difference in OOB error of each feature before and after adding noise to determine the importance of each feature. Thus, the importance of each feature is directly proportional to the difference [54]. We used the RF algorithm to calculate the importance ranking of features in our study.

#### 2.3.4. Fisher Linear Discriminant Analysis (FLDA)

FLDA is a widely used analysis method for classification [17,55]. The basic theory is to project the multidimensional data to a one-dimensional space, so that the distance within the same class is the smallest and the distance between classes is the largest, whereby different classes would have the best segmentation point or line in the space. FLAD has been widely used in plant disease discrimination and classification [31]. FLAD was used in this study to establish an optimal discrimination model and leave-one-out cross-validation was applied to verify classification accuracy. Finally, the value of overall accuracy (OA), producer’s accuracy (P.’s a.), user’s accuracy (U.’s a.), and kappa coefficient were used to evaluate the classification and discrimination performance of FLDA [56]. FLDA was implemented using SPSS 20.0 software (IBM Corporation, New York, NY, USA).

#### 2.3.5. Yellow Rust Mapping Using Optimal Threshold Segmentation

To map disease damage with a single variable, the optimal threshold segmentation method was adopted. Threshold segmentation is a method that separates an image into a number of meaningful regions according to the selected threshold values [57]. For this purpose, 50 evenly spaced intervals were set between the average of each variable. The overall accuracy of each interval value can be figured out based on the validation data. When the overall accuracy comes to highest, the optimal threshold was determined to distinguish different categories [58]. This method is simple in calculation, with high operation efficiency.

## 3. Results

### 3.1. Canopy Spectral Reflectance of Wheat Yellow Rust

#### 3.1.1. Spectral Characteristic of Hyperspectral Data

**Figure 4:**
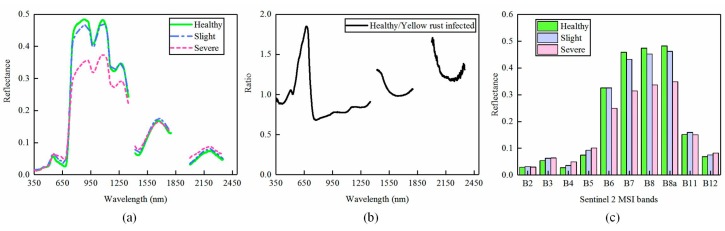
(**a**) The average canopy spectral reflectance of different yellow rust infection levels; (**b**) The spectral ratios of yellow-rust-infected wheat compared to healthy winter wheat; (**c**) The average spectral reflectance of different yellow rust infection levels across the simulated Sentinel-2 spectral bands.

As shown in Figure 4a, the average canopy spectral reflectance of healthy winter wheat and slightly and severely yellow-rust-infected winter wheat is significantly different. The spectral curve of healthy winter wheat has a “green peak” and “red valley” characteristic in the visible (VIS) region, and has a high reflectance in the near-infrared (NIR) region, which is consistent with the typical reflectivity of green vegetation. In the study, the spectral of 1350–1420 nm, 1800–2000 nm, and 2350–2500 nm in SWIR region were removed due to the susceptibility to the strong absorption of water vapor and the weak spectral signal [27]. When compared with the spectrum of healthy samples, winter wheat that is infected with yellow rust disease has different spectral characteristics in the VIS, NIR, and SWIR spectral region due to changes in pigment content, moisture, and canopy architecture. For instance, the red valley of samples infected with yellow rust is raised up in the spectral curve while the reflectance in the near infrared region is decreased when comparing it with healthy samples. Increases and decreases in the reflectance in the red and NIR region are directly proportional to the severity of yellow rust infection. Moreover, the reflectance of diseased samples increases in the shortwave near-infrared (SWIR, 2000–2400 nm) region.

The ratio curve of reflectance, dividing the reflectance of yellow-rust-infected wheat by healthy wheat is shown in Figure 4b. There are three significant differences between the spectral curve of healthy and yellow-rust-infected samples at: 630–800 nm, 1420–1490 nm, 2000–2100 nm, and the most notable difference is located in the spectral region of 640–680 nm. These differences are consistent with previous studies on yellow rust detection [4,5]. The changes in spectral characteristics mentioned above provide a strong theoretical basis for monitoring and discriminating winter wheat yellow rust disease using multispectral satellites.

#### 3.1.2. Spectral Characteristic of Simulated Multispectral Data

Figure 4c shows the mean canopy reflectance of healthy wheat and slightly and severely yellow-rust-infected samples in the simulated Sentinel-2 multispectral data. Healthy samples have a higher reflectance at B6, B7, B8, and B8a (including two red-edge bands) while severely infected yellow rust samples are lower at these bands. Conversely, the reflectance of yellow-rust-infected samples is higher than healthy samples at the B3 (green), B4 (red), B5 (Re1), and B12 (SWIR2). Note that the reflectance derived from simulated multispectral data in visible and NIR region exhibits a similar pattern with the hyperspectral data shown in Figure 4a.

### 3.2. Yellow Rust Disease Discrimination with Optimal Bands

**Figure 5:**
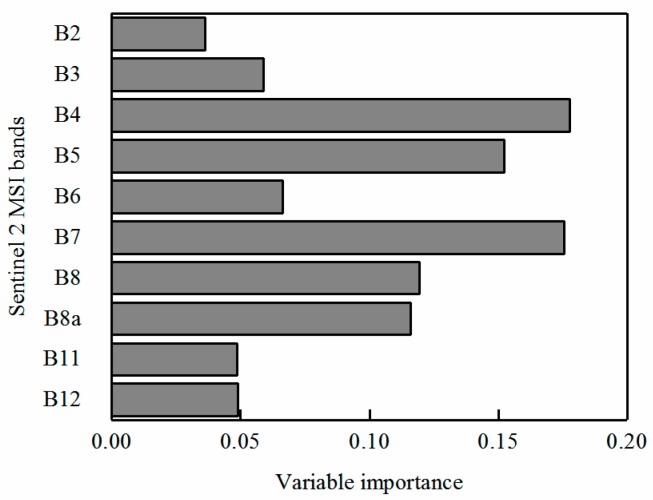
Ranking of Sentinel-2 MSI bands based on their importance for yellow rust discrimination through RF models.

The RF method was used to evaluate the importance of each band in the process of discriminating between healthy wheat and slightly and severely yellow-rust-infected winter wheat, and the ranking of each result is illustrated in Figure 5. The top three most significant bands were B4 (red), B7 (Re3), and B5 (Re1), which implies that B4 was the most sensitive variable for discriminating wheat yellow rust infection, compared with B7 and B5. This occurred due to the chloroplast damage of the disease samples by the yellow rust pathogen resulting in the reflectance of the disease samples having a large shift in the red-edge region of the spectrum compared with healthy samples [8].

### 3.3. A New Index for Identifying Wheat Yellow Rust Disease

#### 3.3.1. Construction of the New Index

**Figure 6:**
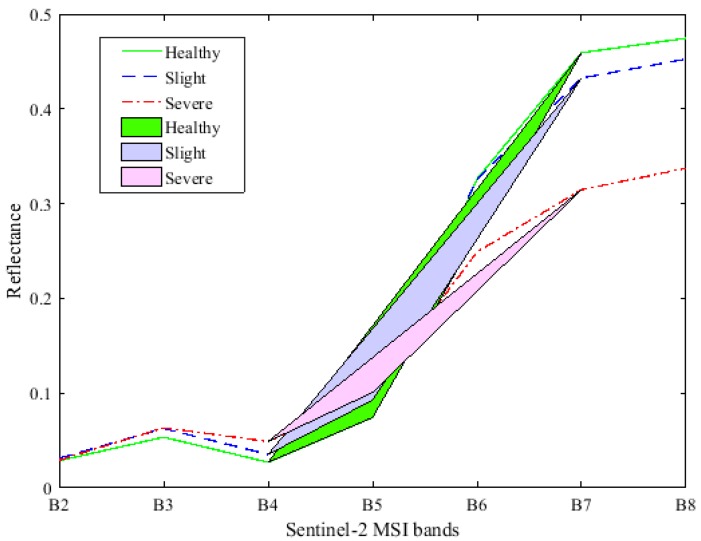
The triangular areas consisting of three sensitive bands under healthy, slight, and severe yellow rust infection; Polyline indicates the average spectral reflectance of different yellow rust infection levels in Sentinel-2 MSI band.

According to the analysis in Section 3.1 and Section 3.2, Red, Re1, and Re3 were the most sensitive bands for identifying winter wheat infected with yellow rust. The reflectance of wheat infected with yellow rust at the red and Re1 bands were higher than that of healthy samples, and there was a positive correlation between the severity of the yellow rust disease and the reflectance of the two bands. Conversely, there was a negative correlation between the reflectance of B7 (Re3, the central wavelength at 783 nm) and the severity of yellow rust infection. This phenomenon was present mainly due to the variation in pigment content and the destruction of cellular structure caused by the yellow rust pathogen. Analysis was based on the function of the Triangle Vegetation Index (TVI), which is defined as the area covered by a triangle in spectral reflectance space based on the reflectance of red, NIR, and green [59]. Therefore, the triangle area defined by B4, B5, and B7 (Red, Re1 and Re3 bands) was the function of the reflectance difference between Re3, Re1, and red, as shown in Figure 6. The formula is given as follows:

(3) AreaTri=(705−665)×(RRe3−RR)−(783−665)×(RRe1−RR)2

In practice, the symptoms of slight yellow rust infection (especially for a DI value of less than 5) in the spectrum are not significantly different from the physiology of healthy wheat, so it is difficult to distinguish slight yellow rust infection from healthy winter wheat when using the triangular area feature. As shown in Figure 6, the greater the severity of yellow rust infection, the smaller the triangle area. Note that the reflectance of the red band increases with the increase in the severity of yellow rust infection. To enhance the difference among the healthy, slightly, and severely yellow-rust-infected winter wheat, the ratio of the triangle area to the reflectance of the red band is calculated to generate a new vegetation index, namely the red-edge disease stress index (REDSI) defined as Equations (4) and (5). The REDSI not only reflects the physiological status of winter wheat infected with yellow rust, but also increases the distinction between healthy and slightly yellow-rust-infected samples. The equations are given as follows:

(4) $$REDSI=\frac{Area_{Tri}}{R_{R}}$$

(5) REDSI=(705−665)×(RRe3−RR)−(783−665)×(RRe1−RR)2×RR

where $Area_{Tri}$ represents the area of the triangle, $R_{i}$ indicates the reflectance of the *i* band (*i* represents red, Re1, and Re3), and the values of 665, 705, 783 represent the central wavelength of the Red, Re1, and Re3 band, respectively.

#### 3.3.2. Classification of Severity of Winter Wheat Yellow Rust Disease

Table 3 summarizes the ability of indexes discriminating between healthy and various levels of yellow-rust-infected winter wheat when using FLDA. REDSI has the highest classification accuracy compared with the other nine sensitive SVIs, including conventional VIs and red-edge VIs; see Section 2.3.2. REDSI achieved the best classification accuracy (up to 84.1%) in the discrimination between healthy and various levels of yellow-rust-infected winter wheat, followed by NREDI1, VARI_green_, and NREDI2 with overall classification accuracies of 81.4%, 79.6%, and 79.6%, respectively (Table 3). REDSI had the highest accuracy compared with other SVIs for monitoring severe yellow-rust-infected winter wheat and its classification accuracy reached to 97.8%. 

The identification ability of REDSI for healthy, slightly, and severely yellow-rust-infected winter wheat was summarized in Table 4. The overall identification accuracy was 84.1% and the kappa coefficient was 0.76. As for healthy and different degrees of yellow rust infection, REDSI produced the highest accuracy to identify severe yellow-rust-infected samples, with a P.’s accuracy of 97.8% and U.’s accuracy of 89.8%, whereas slight yellow rust and healthy samples produced a U.’s accuracy of 80.0% and 79.3%, respectively. The classification error declined with the increasing severity of yellow rust infection. REDSI performed well in identifying healthy and yellow-rust-infected winter wheat on the canopy scale, which indicates that broadband VIs derived from satellite data have potential for identifying and monitoring winter wheat yellow rust diseases.

### 3.4. Mapping Diseases at Regional Scales Using the New Index

We applied REDSI to Sentinel-2 imagery to monitor the yellow rust infection situation in the wheat planting areas of Chuzhou and Hefei cities in order to validate the REDSI’s ability to detect yellow rust infection in winter wheat for different datasets. In the regional survey, the winter wheat samples contained two types of data, namely healthy and yellow-rust-infected wheat. The final output of this study was a distribution map of healthy and yellow-rust-infected winter wheat.

Due to the differences in study areas and spatial scales, we used a stepwise threshold optimization method to find the optimal threshold for REDSI for the detection of yellow rust infection at regional scales [58,60]. The optimal threshold method seeks the best threshold value to split the whole area into healthy and yellow-rust-infected parts to accomplish the detection process. The overall accuracy and kappa coefficient of yellow rust detection were calculated using the validation data obtained from field investigation based on the threshold. The optimal threshold value was defined as the point with the highest identification accuracy.

**Figure 7:**
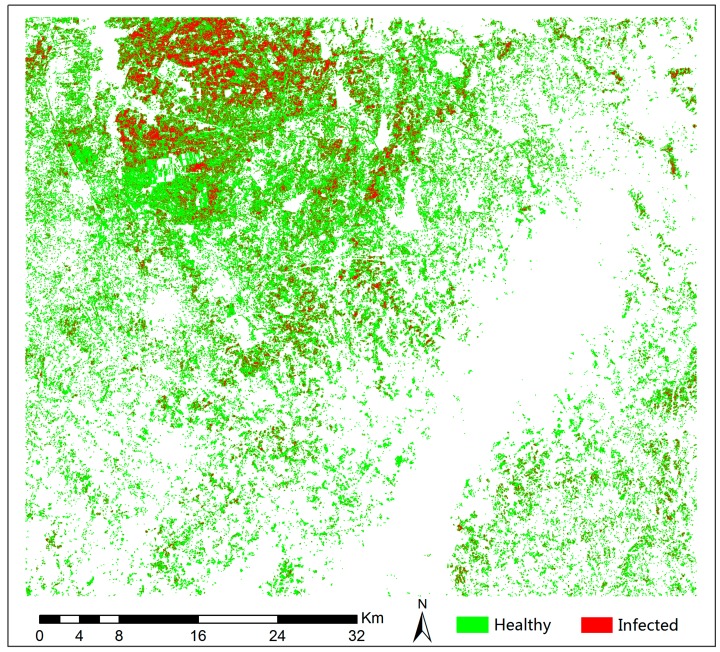
Mapping the spatial distribution of winter wheat yellow rust damage with the Sentinel-2 sensor.

Figure 7 demonstrates the spatial distribution of healthy and yellow-rust-infected winter wheat obtained using the threshold value of 59.7 based on REDSI. The yellow rust infection occurred in most of the area in the yellow rust infection spatial distribution map, especially in the northern part of the study area. The distribution of winter wheat infected with yellow rust was almost the same as the entire winter wheat planting area in the region, while the distribution of yellow rust infection was scattered in the remaining areas. Such a pattern of disease distribution is consistent with the actual occurrence of yellow rust in winter wheat in this region. The confusion matrix in Table 5 shows the classification accuracies for the mapping results after being validated by field investigation data. The optimal threshold method for REDSI achieved good identification results at the regional scale with a kappa coefficient of 0.67 and an overall accuracy of 85.2%. Consequently, REDSI (based on Sentinel-2 MSI data) demonstrated its ability to identify winter wheat yellow rust infection at regional scale.

At regional scale, we also compared the classification ability of REDSI and the commonly used vegetation indexes to detect yellow rust infection using the optimal threshold method. The overall classification accuracy of the new developed REDSI was the best (85.2%) for the healthy and yellow-rust-infected winter wheat, followed by NREDI1, VARI_green_, RGR, and PSRI1, respectively (Table 6). Moreover, the recall clarified that REDSI had higher classification accuracy (87.5%) for identification of healthy wheat, and REDSI and NREDI1 had higher classification accuracy (84.2%) for identification of yellow-rust-infected wheat compared with other common vegetation indexes. Consequently, REDSI (based on Sentinel-2 MSI data) demonstrated its ability to identify winter wheat yellow rust infection at regional scale.

## 4. Discussion

When winter wheat is infected by yellow rust, the leaves begin to wilt and become discolored. Winter wheat presents different physiological symptoms for healthy and diseased canopies depending on the biology of the pathogens and the characteristic host–pathogen interaction [61]. As shown in Figure 3, yellow-rust-infected winter wheat usually has a higher spectral reflectance in the VIS and SWIR region, especially in the red region, and a much lower spectral reflectance in the NIR region compared to healthy winter wheat. Previous studies have indicated that an increase in the reflectance of the VIS region might be associated with a decrease in chloroplast, whereas a lower reflectance in the NIR region is mainly influenced by changes in leaf structure and water content [49,62]. Furthermore, the change of reflectance in the SWIR region is connected with a variation in lignin and protein content [63,64]. All of these spectral features are consistent with the spectral characteristics of simulated Sentinel-2 data in our study and the previous studies of yellow rust on winter wheat [27,35,65], which proves the feasibility and accuracy of using hyperspectral data to simulate Sentinel-2 MSI data.

Our result demonstrates the potential of the red-edge disease stress index (RDESI) for discriminating yellow rust infection in winter wheat based on Sentinel-2 satellite data. The common way to detect significant bands or vegetation index for plant diseases discrimination is by connecting to biochemical or biophysical traits [8]. For example, the yellow rust pathogens can induce changes in biophysical and biochemical parameters of wither wheat, such as variations of several pigments [66], and changes of leaf color due to pustules or lesions [7,67]. These changes will further result in spectral responses, such as the increase of reflectance in a red band and the reduction of reflectance in the near-infrared band. The results of this study have successfully selected the most important bands in the Sentinel-2 MSI (i.e., B4, B5, and B7) for detection of winter wheat with yellow rust infection. This confirms the importance of the spectrum in this region for plant fungal disease detection and discrimination as reported in previous studies. For instance, Devadas et al. have suggested that the most efficient indicator for wheat rust detection is the reflectance of the green to red region [68,69]. Moshou et al. have pointed out that the reflectance of regions at around 705 nm and 725 nm are sensitive to wheat yellow rust detection [4]. The red-edge regions have the ability to evaluate plant stress [9,31]. In Sentinel-2 MSI, B5, B6, and B7 are the red-edge bands and have a strong correlation between each band. According to the principle of RF importance ranking and the results of the calculations [29,31], we choose other bands with no correlation to build the index as possible. B6 was not chosen in the REDSI, which does not mean that B6 is not beneficial for disease detection.

The developed REDSIs are based on the triangle geometry area and the most relevant wavelength ratio regarding wheat yellow rust disease. Triangle geometry area was determined by the reflectance of B4, B5, and B7. The reflectance of B4 and B7 showed the opposite trend under the condition of disease stress. The greater the severity of yellow rust infection, the greater the reflectance of B4, and the smaller the reflectance of B7, which leads to the smaller triangle geometry area. The value the triangle geometry area divided by the reflectance of B4 would increase the separability among healthy, slightly, and severely yellow-rust-infected winter wheat. 

The REDSI produced good results to detect winter wheat yellow rust disease at the canopy and regional scales. At the canopy scale, REDSI’s ability to identify serious yellow rust infection is greater compared with its ability to identify wheat that is healthy and has slight yellow rust infection. It is consistent with the view of Sanakran et al. [66] that the higher the visible symptoms, the better the accuracy of disease detection. In addition, the leaves of severe yellow rust infection samples covered with more pustules would increase the spectral separability between the healthy and slight infection samples due to the significant difference in leaf color [34]. The optimal threshold method is used with REDSI for detecting yellow rust disease at the regional scale in practical applications. The optimal threshold method provides an efficient and simple disease mapping when compared with other complex methods (e.g., support vector machine, neural networks, and so on). It should be clear that the threshold value is not a constant value and may be slightly different from crop cultivars, experiment time, or study areas. Due to the low number of severe yellow rust ground truth data, the mapping of winter wheat yellow rust was divided into healthy and yellow-rust-infected categories at the regional scale. More samples of different severities of yellow rust infection need to be collected to test the feasibility of REDSI at the regional scale in future. It is important to detect and map the infections of plant diseases in the field. The distribution result of plant infection in the field could provide useful information for decision making on the necessity and appropriate timing of precise fungicide applications. It not only improves productivity, but also mitigates field contamination. In summary, REDSI’s identification accuracy is able to meet the practical demands for yellow rust disease detection, and has demonstrated its potential (using Sentinel-2 imagery) as a valuable index for detecting yellow rust disease in winter wheat. More importantly, Sentinel-2 provides a frequent revisit cycle of about 10 days (or 5 days if two satellites operate at the same time). It provides a fast, non-destructive, effective, and labor-saving approach for identifying and monitoring yellow rust disease at regional scales, which is beneficial for field management and the development of the agricultural insurance business.

In the study, REDSI can be used for yellow rust monitoring using independent datasets from different measuring devices and spatial scales, which demonstrates the robustness and potential of REDSI for yellow rust discrimination in practice. However, it should be noted that not only yellow rust disease stress but other diseases (e.g., powdery mildew, Fusarium head blight) may occur simultaneously in the field during winter wheat growth stages. So, future work will be needed to explore REDSI’s ability to identify and monitor other winter wheat diseases. In addition, the environmental conditions are much more complicated in practice, and the occurrence of disease is also closely related to biotic conditions and the ecological environment, including: temperature, humidity, meteorological conditions, plant density, and field management strategies. For example, Danial and Parlevliet suggest that the density of vegetation can affect the severity levels of yellow rust [70]. Therefore, these factors should be considered in future work when attempting to acquire a high identification accuracy at regional scales.

## 5. Conclusions

In this study, we used canopy hyperspectral data to simulate the corresponding multispectral bands of Sentinel-2, based on the RSR function of the Sentinel-2 multispectral sensor, and developed a new index, REDSI (consisting of Red, Re1, and Re3 bands), for detecting and monitoring yellow rust infection of winter wheat at the canopy and regional scale. Compared with other common spectral vegetation indexes, REDSI has excellent performance in detecting and monitoring yellow rust in winter wheat at the canopy and regional scale, with the overall accuracy of 84.1% and 85.2%, respectively. Furthermore, the index had to be continually validated with other diseases and other cultivars to guide agriculture precision management.

## Figures and Tables

**Table 1:** The spectral bands and resolutions of Sentinel-2 MSI sensor.

Spectral Band		Centre Wavelength (nm)	Band Width (nm)	Spatial Resolution (nm)
B1	Coastal aerosol	443	20	60
B2	Blue (B)	490	65	10
B3	Green (G) ^1^	560	35	10
B4	Red (R) ^1^	665	30	10
B5	Red-edge 1 (Re1) ^1^	705	15	20
B6	Red-edge 2 (Re2) ^1^	740	15	20
B7	Red-edge 3 (Re3) ^1^	783	20	20
B8	Near infrared (NIR) ^1^	842	115	10
B8a	Near infrared narrow (NIRn) ^1^	865	20	20
B9	Water vapor	945	20	60
B10	Shortwave infrared/Cirrus	1380	30	60
B11	Shortwave infrared 1 (SWIR1)	1910	90	20
B12	Shortwave infrared 2 (SWIR2)	2190	180	20

^1^ indicates *p*-value < 0.001.

**Table 2:** Summary of spectral vegetation indexes used for detecting yellow rust.

SVIs	Definition	Formula	Reference
Conventional Vis			
NDVI	Normalized difference vegetation index	$\left(R_{NIR}-R_{R})/(R_{NIR}+R_{R}\right)$	[46]
EVI	Enhanced vegetation index	$2.5(R_{NIR}-R_{R})/(R_{NIR}+6R_{R}-0.5R_{B}+1)$	[52]
RGR	Ration of red and green	$R_{R}/R_{G}$	[47]
VARI_green_	Visible atmospherically resistant index	$\left(R_{G}-R_{R})/(R_{G}+R_{R}\right)$	[49]
Red-edge vegetation indexes			
NDVIre1	Normalized difference vegetation index red-edge1	(RNIR−RRe1)/(RNIR+RRe1)	[46]
NREDI1	Normalized red-edge1 index	(RRe2−RRe1)/(RRe2+RRe1)	[50]
NREDI2	Normalized red-edge2 index	(RRe3−RRe1)/(RRe3+RRe1)	[50]
NREDI3	Normalized red-edge3 index	(RRe3−RRe2)/(RRe3+RRe2)	[50]
PSRI1	Plant senescence reflectance index	(RR−RG)/RRe1	[42,53]

**Table 3:** Comparison of the REDSI’s classification ability with other SVIs.

Index	Classification Accuracy (%)	Recall		
		Healthy (%)	Slight (%)	Severe (%)
REDSI	84.1	79.3	71.8	97.8
VARI_green_	79.6	86.2	61.5	91.1
RGR	77.0	86.2	59.0	86.7
EVI	68.1	58.6	48.7	91.1
NDVI	78.8	89.7	64.1	84.4
PSRI1	77.9	82.3	59.0	91.1
NREDI1	81.4	86.2	69.2	88.9
NREDI3	74.3	89.7	79.5	60.0
NREDI2	79.6	86.2	74.4	80.0
NDVIre1	78.8	86.2	71.8	80.0

**Table 4:** A confusion matrix and the classification accuracies of the REDSI discriminant model for identifying healthy and yellow-rust-infected wheat.

REDSI	Healthy	Slight	Severe	Sum	U.’s a (%)	OA (%)	Kappa
Healthy	23	6	0	29	79.3	84.1	0.76
Slight	6	28	1	35	80		
Severe	0	5	44	49	89.8		
Sum	29	39	45				
P.’s a (%)	79.3	71.8	97.8				

**Table 5:** Confusion matrix and classification accuracies calculated from field survey data.

	Healthy	Infected	Sum	U.’s a (%)	OA (%)	Kappa
Healthy	7	3	10	70.0	85.2	0.67
Infected	1	16	17	94.1		
Sum	8	19				
P.’s a (%)	87.5	84.2				

**Table 6:** Comparison of the REDSI’s classification ability with other SVIs at the regional scale.

Index	Overall Classification Accuracy (%)	Recall	
		Healthy (%)	Infected (%)
REDSI	85.2	87.5	84.2
VARI_green_	74.1	62.5	78.9
RGR	74.1	62.5	78.9
EVI	66.7	50.0	73.7
NDVI	66.7	50.0	73.7
PSRI1	74.1	62.5	78.9
NREDI1	81.5	75.0	84.2
NREDI3	66.7	50.0	73.7
NREDI2	74.1	62.5	78.9
NDVIre1	74.1	62.5	78.9
